# A Spectroscopic Study on the Amyloid‐β Interaction with Clicked Peptide‐Porphyrin Conjugates: a Vision Toward the Detection of Aβ Peptides in Aqueous Solution

**DOI:** 10.1002/cbic.202400431

**Published:** 2024-11-07

**Authors:** Rita Tosto, Stefania Zimbone, Giuseppina Sabatino, Giuseppe Di Natale, Maria Laura Giuffrida, Marianna Flora Tomasello, Luca Lanzanò, Tiziana Campagna, Sonia Covaceuszach, Graziella Vecchio, Giuseppe Pappalardo

**Affiliations:** ^1^ Institute of Crystallography National Research Council Via Paolo Gaifami 18 95126 Catania Italy; ^2^ International PhD School of Chemical Sciences Department of Chemical Sciences University of Catania Viale Andrea Doria, 6 95125 Catania Italy; ^3^ Department of Physics and Astronomy University of Catania Viale Andrea Doria, 6 95125 Catania Italy; ^4^ Institute of Crystallography National Research Council Strada Statale 14 Km 16.5 1434149 Basovizza (TS) Italy; ^5^ Department of Chemical Sciences University of Catania Viale Andrea Doria, 6 95125 Catania Italy

**Keywords:** Alzheimer's Disease (AD), Amyloid-β (Aβ), Amyloid sensor, Circular Dichroism (CD), Porphyrins, Grating-Coupled Interferometry (GCI)

## Abstract

Alzheimer's disease (AD) is a multifactorial form of dementia mainly affecting people in the elderly, but no effective cure is available. According to the amyloid hypothesis the aggregation of Amyloid‐β (Aβ) into oligomeric toxic species is believed to concur with the onset and progression of the disease heavily. By using a click chemistry approach, we conjugated a suitable designed peptide sequence to a metalloporphyrin moiety to obtain three hybrid peptide systems to be studied for their interaction with Amyloid‐β peptides. The aim is to get new tools for the diagnosis and therapy in AD. The results described in this study, which were obtained through spectroscopic techniques (UV‐Vis, CD, bis‐Ans and intrinsic porphyrin Fluorescence), Microfluidics (GCI) and cell biology (MTT, Live cell imaging and flow cytometry), reveal interesting features about the structure‐activity relationships connecting these conjugates with the interaction with Aβ, as well as on their potential use as sensing systems. In our opinion the data reported in this paper make the porphyrin‐peptide conjugates highly compelling for further exploration as spectroscopic probes to detect Aβ biomarkers in biological fluids.

## Graphical abstract

Three porphyrin‐peptide conjugates have been synthesized by copper catalyzed azide‐alkyne cycloaddition (CuAAC) and their interaction with Aβ42 investigated using different experimental techniques. The zinc containing peptide conjugate can evoke an induced dichroic signal upon its interaction with Aβ42. The peptide systems are not cytotoxic and can be taken up by differentiated SH‐SY‐5Y cells.

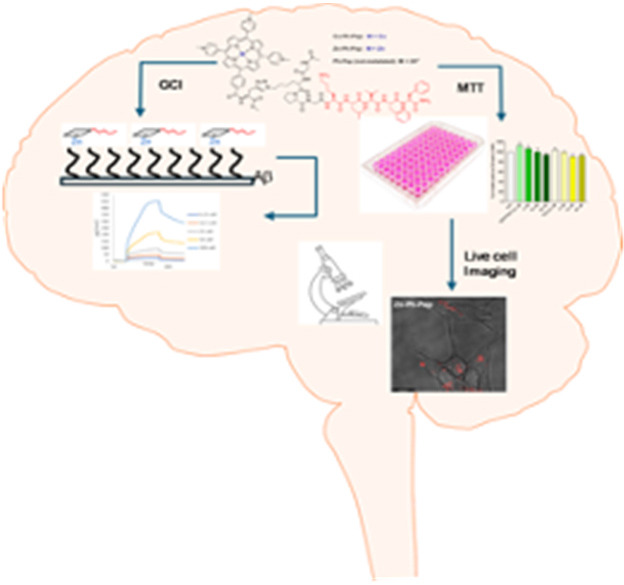

## Introduction

Amyloid aggregation is associated with a variety of pathological conditions including systemic amyloidosis and neurodegeneration.^[1]^ Alzheimer's Disease (AD) is the most common neurodegenerative disease in humans accounting for more than 47 million cases worldwide. This number is expected to reach 132 million people affected by 2050, according to the World Health Organization (WHO) and Alzheimer Disease International (ADI) estimations, in turn producing an enormous burden on the healthcare systems of developed countries.^[2]^ AD is a progressive and irreversible pathology without any effective cure. For this reason, early or even preventive interventions seem to be a promising strategy to alleviate AD symptoms. Several strategies have been considered, most of them aimed at inhibiting the Aβ peptide aggregation pathways, by stabilizing the physiologically active monomeric form of the peptide.[^3^, ^4^, ^5^] Other approaches aimed at regulating the Aβ production and accumulation,^[6]^ as well as plaque dissolution.^[7]^ The oligomeric forms of Aβ are directly responsible for the neurotoxic effects observed in AD. Therefore, it is important to expand the collection of molecular systems capable of intervening and blocking the early events of the Aβ aggregation process by preventing the recruitment of the Aβ monomer into self‐assembled toxic forms. The prevention of the Aβ aggregation process usually implies the direct interaction of the inhibitor with the amyloid peptide that can exist in different aggregation states.^[8]^ Thus, the knowledge of the structural properties of Aβ in the various aggregation states may facilitate the design and/or the discovery of molecules that can specifically interfere with Aβ‐Aβ interaction and assembling.^[9]^ Peptides are excellent candidates for this task. Indeed, the developments in peptide molecular biology and synthetic protocols for their chemical modifications spread the use of peptide‐based therapeutics and diagnostics.^[10]^ In particular, the conjugation of small peptides with functional prosthetic groups offers unique molecular tools endowed with multiple activities, especially at the diagnostic and/or therapeutic levels.[^11^, ^12^, ^13^, ^14^, ^15^, ^16^, ^17^, ^18^] In this regard, experimental procedures employing porphyrin‐peptide conjugates for application in photodynamic therapy (PDT) are well documented.[^19^, ^20^, ^21^, ^22^] Yet, the use of conjugated peptide systems is becoming increasingly popular also for a diagnostic and/or therapeutic approach to AD.[^23^, ^24^, ^25^, ^26^, ^27^, ^28^] We have been dealing with the synthesis of peptide conjugates designed as inhibitors of Aβ’s aggregation for a long time.[^14^, ^29^, ^30^, ^31^, ^32^] Among these, we have previously reported the neuroprotective and anti‐fibrillogenic abilities of a porphyrin‐peptide conjugate bearing the GPGKLVFF moiety.[^31^, ^32^] Porphyrins possess interesting photophysical, chemical, and biological properties making them versatile molecular platforms in a wide range of applications.[^33^, ^34^, ^35^, ^36^] Particularly, metalloporphyrins’ anti‐aggregation ability toward amyloid peptides has been documented.[^37^, ^38^, ^39^, ^40^]

In this work we describe the synthesis and the interaction with Aβ42 of three new conjugates with the meso‐tri(N‐Methyl‐4‐Pyridyl) mono(4‐carboxyphenyl) Porphine tri‐chloride (MCPTPyP or ***Ph***) covalently linked via click chemistry to a suitable peptide, containing the KLVFF sequence. The interaction with Aβ42 was studied by different spectroscopic techniques including UV‐Vis, CD, and fluorescence, while the binding constants (comprising the affinity constant KD and kinetic constants K_on_ and K_off_) were measured by means of the Grating‐Coupled Interferometry (GCI) technique. Finally, we carried out preliminary experiments to test any potential cells toxicity associated with these peptide constructs alongside their ability to enter the cells. In this regard, we performed 3‐(4,5‐Dimethylthiazol‐2‐yl)‐2,5‐Diphenyltetrazolium Bromide MTT assay, flow cytometry and confocal microscopy imaging to respectively verify these occurrences.

## Results

### Design and Synthesis of the Porphyrin‐Peptide Conjugates

We previously described the synthesis of a novel meso‐substituted tricationic metalloporphyrin‐peptide conjugate in which the carboxyphenyl group at mesoposition of the porphyrin core is linked via an amide bond to the N‐terminal amino function of the short peptide containing the well‐known hydrophobic core Aβ_16–20_ (KLVFF). This metalloporphyrin‐peptide conjugate showed amyloid inhibition properties, while preserving neurons from the cytotoxic effects of β‐amyloid's oligomers.^[31]^

**Scheme 1:**
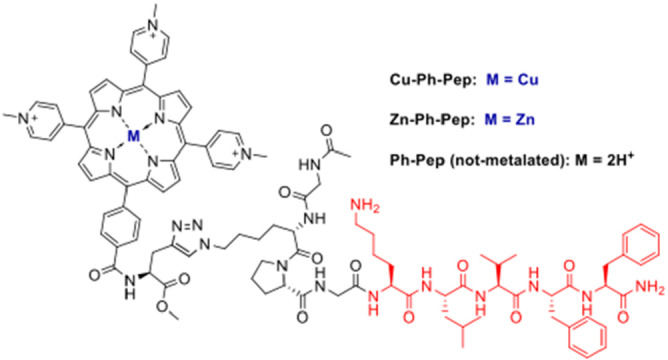
Copper and zinc porphyrin‐peptide conjugates.

In prosecution of our studies on this argument, we herein report new porphyrin‐peptide conjugates obtained by the copper catalyzed azide‐alkyne cycloaddition (CuAAC), the widely used “click” reaction that represents one of the most explored reactions to couple biomacromolecules, easily carried out under ambient or mild conditions in organic or aqueous solvents.^[11]^ The mild condition of the CuAAC reaction is compatible with the peptide chemistry and offers an opportunity to synthesize new peptide conjugates for different applications.^[11]^ We carried out CuAAC reactions, both in solution and on resin, to obtain three porphyrin‐peptide conjugates, two of them differing in the metal ion (Cu or Zn) inserted within the porphyrin's macrocycle, while the third variant lacked any metal (Scheme 1).

**Scheme 2:**
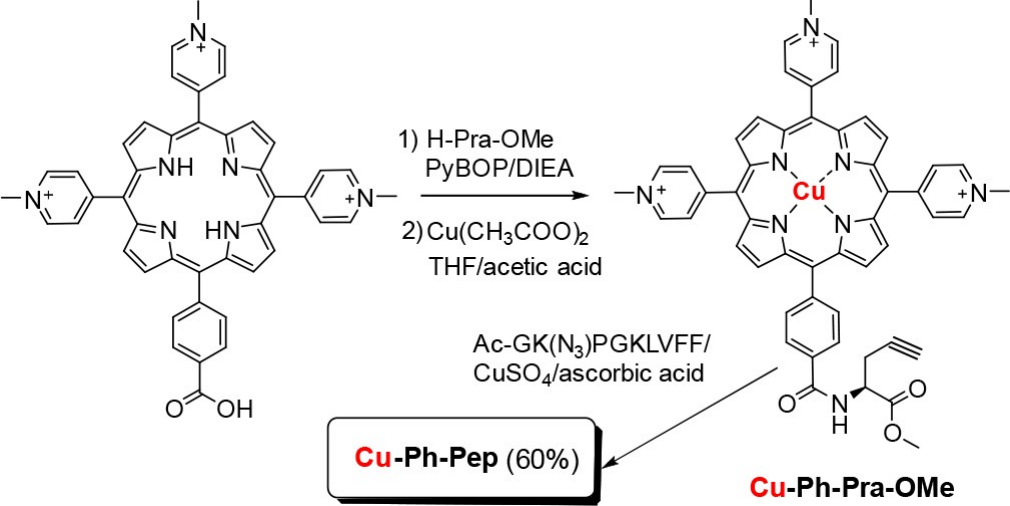
Synthesis of the copper porphyrin‐peptide conjugate ***Cu‐Ph‐Pep***.

The alkynyl group, necessary to perform the CuAAC reaction, was linked to the porphyrin ring by coupling the carboxy phenyl group of the porphyrin core and the N(alpha)‐L‐propargylglycine methyl ester (L‐PraOMe). After testing different activators and reaction condition, the best result was obtained using PyBOP and DIEA in DMF (Scheme 2).

The azido peptide Ac‐GK(N3)PGKLVFF‐NH_2_, was synthetized by 9‐fluorenylmethoxycarbonyl (Fmoc)/tBu automatic microwave‐assisted solid‐phase peptide synthesis (MW‐SPPS).

**Scheme 3:**
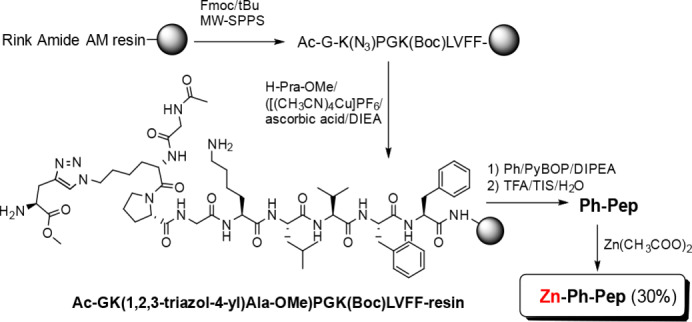
Synthesis of the Zn porphyrin‐peptide conjugate (***Zn‐Ph‐Pep***). The corresponding unmetalated ***Ph‐Pep*** analogue was obtained by taking apart a suitable aliquot of porphyrin‐conjugate peptide before zinc introduction.

The conjugation of Cu−Ph‐Pra‐OMe to the peptide Ac‐GK(N3)PG‐KLVFF‐NH_2_ by the CuAAC reaction was optimized modifying solvent and temperature. The best result was obtained with CuSO_4_/ascorbic acid (1 : 10) in DMF at 50 °C for 24 h followed by an incubation at room temperature for 3 days (Scheme 3). In this condition, the desired product ***Cu−Ph‐Pep*** was obtained, yielding 60 % in a single step. Because the copper species required for the CuAAC reaction can form an unwanted copper‐porphyrin complex, the Zn porphyrin‐peptide conjugate and the unmetalated derivative were obtained using a different approach. We first carried out the CuAAC between the Pra‐OMe and the azido group of the Ac‐GK(N3)PGK(Boc)LVFF peptide still anchored on the solid support (Scheme 3)

The porphyrin was attached to the fully protected peptide on resin, via a selective amide bond formation between the carboxy phenyl group at the meso‐position of porphyrin and the alpha‐amino group of the Pra‐OMe (Scheme 3).

### Spectroscopic Characterization of the Porphyrin‐Peptide Conjugates

**Figure 1:**
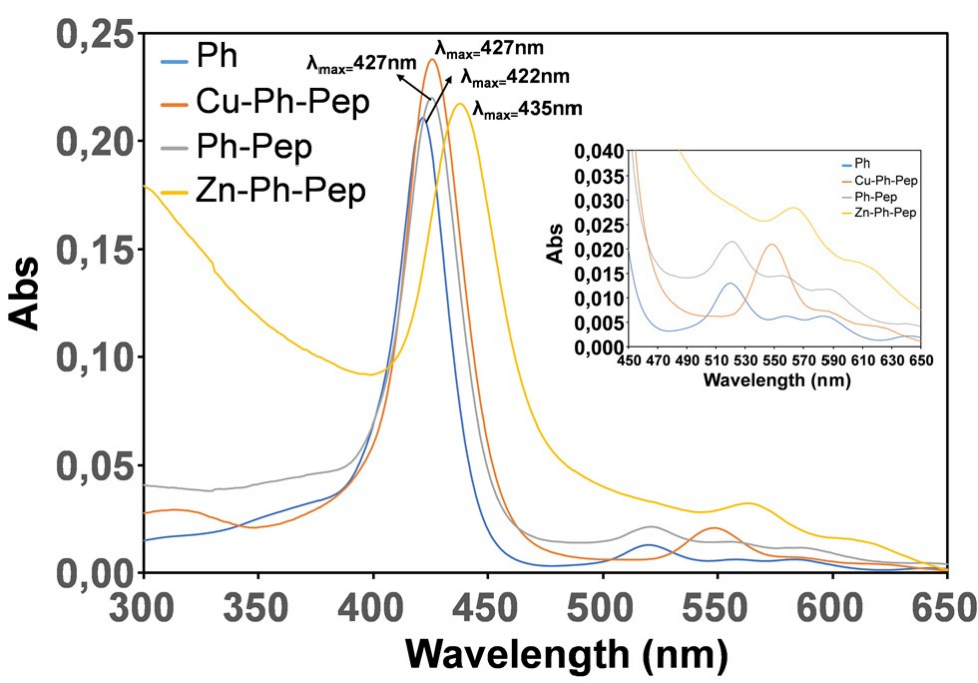
UV‐Vis spectra acquired at different incubation times, in the region between 300–650 nm.

We first studied the effects of peptide conjugation on the porphyrin chromophore, by UV‐Vis spectra in the region between 300–650 nm. The spectra were recorded using a solution of 5 μM Porphyrin‐peptide conjugate in 10 mM phosphate buffer pH 7.4. The relative spectra are shown in Figure 1. The UV‐Vis profile of the porphyrin precursor MCPTPyP, recorded at the same concentration, is also shown for the sake of comparison (Figure 1).

**Figure 2:**
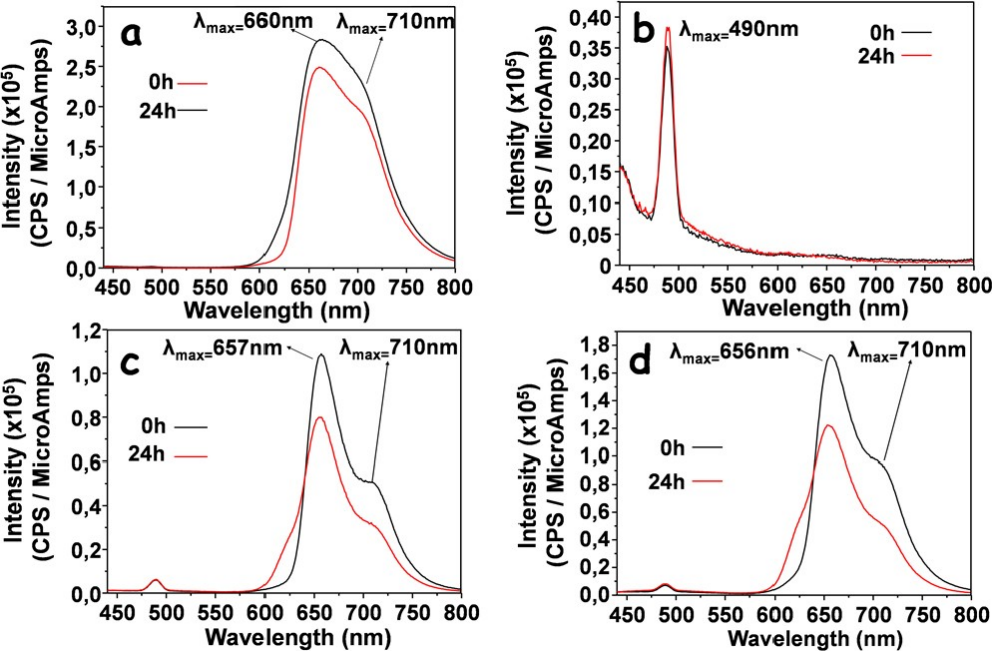
Fluorescence spectra of a) ***Ph***; b) ***Cu‐Ph‐Pep***; c) ***Ph‐Pep***; d) ***Zn‐Ph‐Pep***, recorded a t=0 h and t=24 h. *The signal at around 490 nm can be tentatively assigned to the Raman valence band of OH‐groups from water.^[42]^

Peptide conjugation influences the spectral features of the porphyrin chromophore as evidenced by the red shift in the absorption maximum in the region of the Soret band region. The Q bands are also affected, particularly in the ***Cu‐Ph‐Pep*** and ***Zn‐Ph‐Pep*** compounds (Figure 1). In this case the presence of a metal ion within the porphyrin's core might contribute to the observed spectral profile of these compounds. It is important to point out that at 5 μM, no significant changes in the UV‐Vis intensities and shape were observed in the 0–120 h interval of time for the ***Ph‐Pep*** and ***Cu‐Ph‐Pep*** derivatives. This may suggest the absence of significant self‐aggregation phenomena in these experimental conditions. By contrast, the ***Zn‐Ph‐Pep*** conjugate, exhibits a slight propensity to self‐aggregation as revealed by the variability of the spectra intensities associated with baseline scattering (Figure 1S). The fluorescence spectra of the investigated systems were also acquired in view of the interaction of the studied conjugates with cells (see below biological section).^[41]^ The fluorescence spectra were recorded with the photoexcitation at 425±5 nm, in 10 mM phosphate buffer pH 7.4 and 5 μM concentration. The emission spectrum of the porphyrin precursor MCPTPyP (***Ph***) was also obtained for the sake of comparison. This exhibits an intense and broad emission band around 660 nm along with a weaker shoulder above 710 nm (Figure 2, Panel a).

Detectable changes, in the shape and intensity of the emission bands, were observed for the peptide‐porphyrin conjugates compared to the pristine MCPTPyP. As shown in Figure 2, the emission intensities of the ***Zn‐Ph‐Pep*** and ***Ph‐Pep*** compounds are somewhat decreased, exhibiting a slight blue‐shift of the emission maxima and a pronounced shoulder. In our hands, the derivative ***Cu‐Ph‐Pep*** is not fluorescent.^[43]^

### The Interaction of the Porphyrin‐Peptide Conjugates with Aβ42

The KLVFF peptide can recognize and co‐assemble with full‐length Aβ through hydrogen bonding and hydrophobic interactions.[^44^, ^45^] As demonstrated in our previous studies, the porphyrin. macrocycle can strengthen these interactions.[^31^, ^32^] In the present work we employed UV‐Vis, CD spectroscopy and Bis‐Ans fluorescence to explore the molecular processes underlying the interactions occurring between Aβ and the porphyrin‐peptide conjugates.

**Figure 3:**
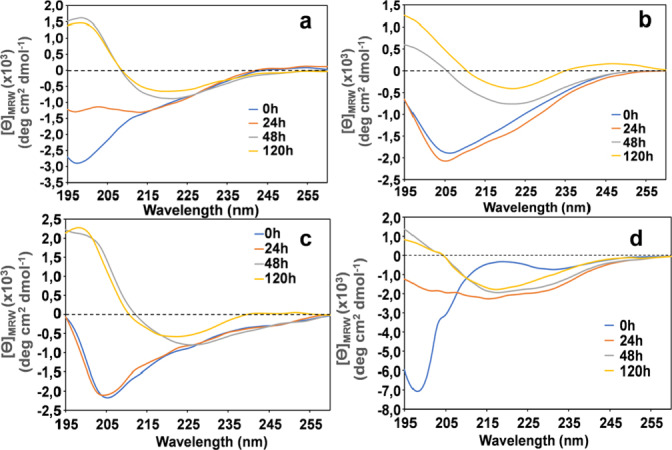
a) CD Spectra of 5 μM Aβ42 recorded at different time intervals in 10 mM phosphate buffer pH 7.4. b–d) CD difference spectra of Aβ42 in the presence of equimolar ration of conjugates: b) ***Cu‐Ph‐Pep***; c) ***Ph‐Pep***; d) ***Zn‐Ph‐Pep***.

The UV‐Vis spectra of porphyrin‐peptide conjugates (5 μM) were recorded in the presence of an equimolar ration of Aβ42 monomers at 0, 24, 48 and 120 h (Figure 2S). The quick comparison with the Uv‐Vis spectra reported in Figure 1S for the pure compounds, reveals minor variations in the spectral profiles for both the Aβ42/***Cu‐Ph‐Pep*** (Figure 2S, Panel a) and the Aβ42/***Ph‐Pep*** (Figure 2S, Panel b) binary systems, with an observable absorbance diminution for the Aβ42/***Cu‐Ph‐Pep*** mixture at 120 h. By contrast, the decreased absorption intensity of the spectra acquired for the Aβ42/***Zn‐Ph‐Pep*** system may suggest a rapid formation of aggregated species in the sample mixture. β‐sheet conformational transition of the Aβ peptide chain is a crucial step leading to fibril formation in the amyloidogenic process.^[46]^ Therefore, the interaction of the peptide‐porphyrin conjugates with Aβ was further studied using CD spectroscopy. The Aβ42 aggregation process was monitored either in the absence or in the presence of an equimolar amount of the conjugated peptides. A series of CD control experiments were preventively carried out to assess the propensity of the porphyrin‐peptide conjugates to adopt a β‐sheet conformation. The corresponding CD curves are shown in the supplementary material (Figure 3S). The observed CD profiles suggest that ***Ph‐Pep*** and ***Cu‐Ph‐Pep*** derivatives can fairly adopt β‐sheet conformation over time, the ***Zn‐Ph‐Pep*** being faster in this process (see Figure 3S). We then evaluated the ability of the porphyrin‐peptide conjugates to affect the Aβ42’s aggregation process. This was accomplished by running a series of CD experiments on the binary systems in the same experimental conditions as the control CD measurements. The CD spectra are shown in Figure 3. A 5 μM Aβ42 sample was used as a positive control of the aggregation process (Figure 3, panel a). In this case, the curve profiles indicate that at 0 h, Aβ42 is predominantly in the random coil conformation, as evidenced by the negative ellipticity around 200 nm. The CD curve observed at 48 h shows that the transition toward β‐sheet conformation is almost completed. The typical dichroic profile characterized by a negative ellipticity at λ=222 nm and a positive band at λ=200 is indicative of this event (Figure 3, panel a).

Figure 3, panels b–d, shows the CD profiles of the Aβ42/porphyrin‐peptide binary systems corrected by subtracting the respective porphyrin‐peptide conjugate background from the experimental spectra of the mixture (not shown). This allows to isolate the changes in the Aβ42 conformation. The resulting CD profiles indicate that Aβ42 retained its random coil status up to 24 h; after this time, none of the peptide conjugates were able to inhibit Aβ42 from adopting the β‐sheet conformation.

**Figure 4:**
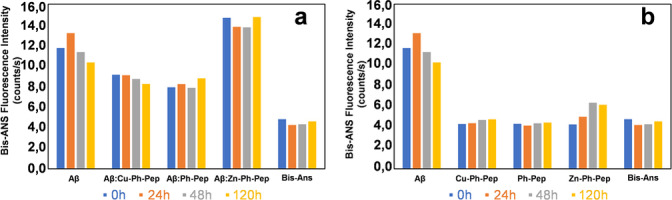
bis‐Ans Fluorescence intensities acquired for the a) binary systems Aβ42/***Cu‐Ph‐Pep***; Aβ42/***Ph‐Pep***; Aβ42/***Zn‐Ph‐Pep***; b) ***Cu‐Ph‐Pep***; ***Ph‐Pep***; ***Zn‐Ph‐Pep***; in the absence of Aβ42.

We also carried out fluorescence assays to evaluate the anti‐fibrillogenic ability of the porphyrin‐peptide conjugates. We used the bis‐Ans (4,4’‐Dianilino‐1,1’‐binaphtyl‐5,5’disulphonic acid) as a chromophore instead of the typical Th−T (Thioflavin‐T), to avoid any potential interference that might occur between the porphyrin and Th−T, in both the excitation and emission wavelength regions. The bis‐Ans probe is sensitive to Aβ fibril formation, and it specifically binds to solvent‐exposed hydrophobic surfaces leading to an increase in its fluorescence emission maximum at 510 nm, in our case.^[47]^ The bis‐Ans fluorescence kinetics were recorded with Aβ42 (20 μM) alone or in the presence of the porphyrin‐peptide conjugates at a 1 : 1 molar ratio. The kinetic profiles of the porphyrin‐peptide conjugates in the absence of Aβ42 were also acquired for comparison (Figure 4S). Figure 4 shows the histogram representation of the bis‐Ans fluorescence intensities at 510 nm collected over the same time interval for each peptide conjugate both in the absence and in the presence of Aβ42. The obtained fluorescence intensities were compared with the values obtained for Aβ42 alone.

Consistent with the CD data, the bis‐Ans fluorescence measurements, acquired for Aβ42 in the presence of the conjugates, demonstrated their ability to interfere with the fibrillogenic process of Aβ42. However, they were unable to completely prevent Aβ42 to form, amyloid fibrils. Notably, the enhancement in bis‐Ans fluorescence observed in the Aβ42/***Zn‐Ph‐Pep*** pair suggests that ***Zn‐Ph‐Pep*** might support or even accelerate Aβ42’s fibril formation (Figure 4, Panel a and Figure 4S Panel c).

### Aβ42 Sensing by Grating‐Coupled Interferometry and CD Measurements

**Figure 5:**
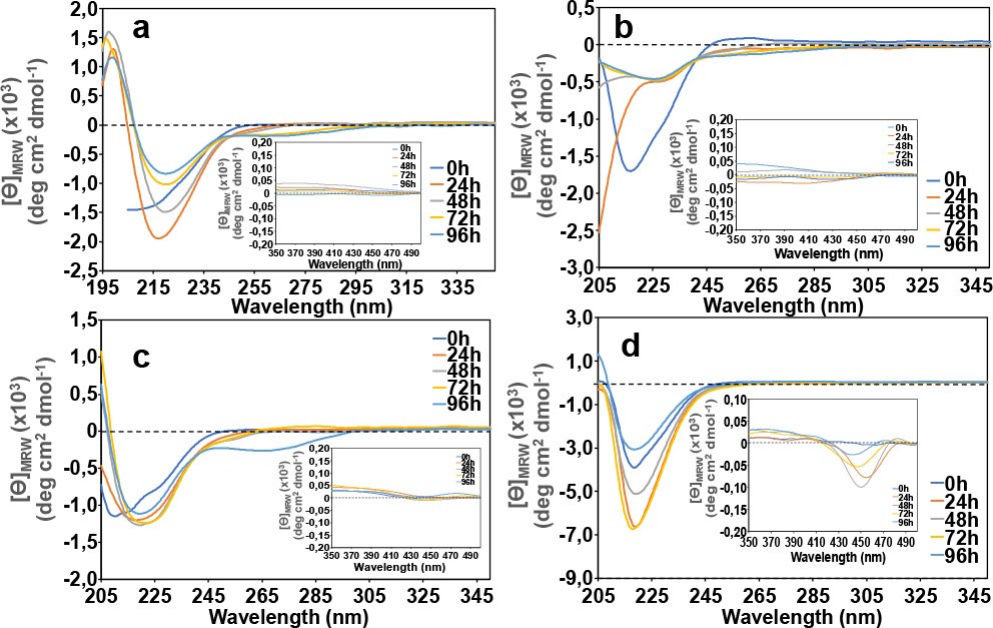
a) CD Spectra of Aβ42 (20 μM) in the Far‐UV region (195–350 nm) and near‐UV region (350–500 nm, inset). CD Spectra of Aβ42 in the presence of the porphyrin‐peptide conjugates (1 : 1 molar ratio) in the Far‐UV region (195–350 nm) and in the near‐UV region (350–500 nm, inset). b) ***Cu‐Ph‐Pep***; c) ***Ph‐Pep***; d) ***Zn‐Ph‐Pep***. The signal at t=0 h was recorded in the interval 200–350 nm because below 200 nm the HT value was too high, and a distorted signal would be obtained (panel a).

**Figure 6:**
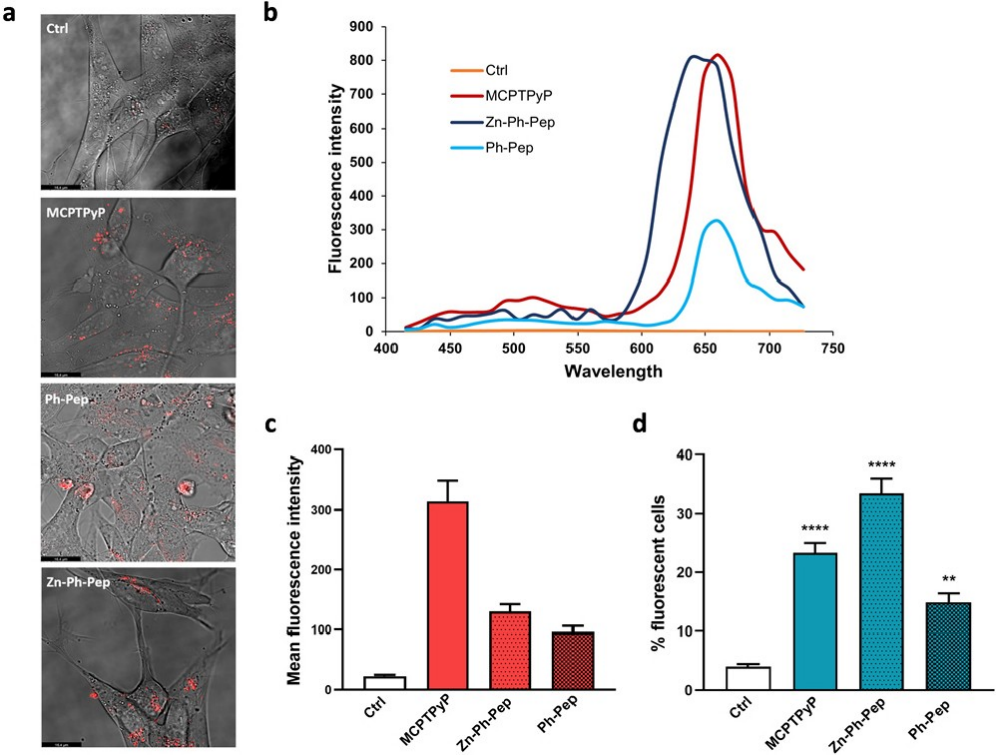
a) Representative picture of the merge between transmitted light images showing the morphology of the cells, and fluorescent images showing the distribution of the fluorescent signal (600–700) recorded inside cells. b) Fluorescence spectra measured for each compound inside cells. Excitation was obtained by using the 405 nm laser and the emissions spectra for each compound were collected by using the lambda scan function. Specifical signals were detected for all sample between 600 nm and 700 nm. c) Histograms show the average fluorescence intensity of cells exposed to 5 μM the above indicated compounds for 24 h. The fluorescence intensity was quantified considering regions of interest (ROI) inside each cell and extracting the average ROI value after background subtraction. d) Histograms show the percentage of fluorescent cells exposed to 5 μM of the above indicated compounds for 24 h. The % of fluorescent cells in FL4 above the established threshold was measured by flow cytometry as described in detail in the experimental section. Bars represent means±SEM of three independent experiments performed in triplicate. Statistically significant differences are indicated with ****P<0.0001 vs Ctrl by One‐Way ANOVA+Tukey's test.

The Grating‐Coupled Interferometry (GCI) is a widely used analytical technique for characterizing intermolecular interactions. It allows quantitative measurements of both the affinity and the kinetics of the binding events. This high sensitivity label‐free method was exploited to measure the interactions of Aβ42 with ***Cu‐Ph‐Pep***, ***Ph‐Pep***, and ***Zn‐Ph‐Pep*** (Figure 5S). Control experiments with pristine MCPTPyP and the peptide GPGKLVFF (***Pep***) were carried out for comparison. The calculated association and dissociation kinetic constants (k_on_ and k_off_, respectively) and the thermodynamic binding constant (KD), are summarized in Table 1. The obtained values indicate that all the conjugates show good affinities with Aβ42. It is also clear from Table 1 that the peptide moiety is fundamental for the molecular recognition of the Aβ42 peptide.^[48]^ Indeed, compared to the pristine porphyrin, the conjugated peptides demonstrate a significantly enhanced efficiency in binding to Aβ42. In addition, the binding affinity of the peptide alone is not significantly different from that of the conjugated derivatives. In fact, the peptide alone may exhibit slightly better affinity, likely because the porphyrin moiety could introduce some steric hindrance, that slightly affects the interaction. Nevertheless, it is important to emphasize that the interaction with Aβ42 is primarily mediated by the peptide rather than by the porphyrin macrocycle. We next investigated whether these peptide derivatives could be employed as molecular probes for the detection of Aβ42 aggregated species. A new set of CD experiments was then carried out with Aβ42 at 20 μM both in the absence and in the presence of the porphyrin‐peptide conjugates at 1 : 1 molar ratio in the UV (195–350) and Visible (350–500) regions. We were expecting that at t=0 h, the CD spectra of Aβ42 (Figure 5, panel a) exhibited broad negative ellipticity over 200 nm suggesting the presence of a mixture of random coil and β‐sheet structures. The signal rapidly evolved toward the typical β‐sheet profile (already within 24 h) with a single minimum at 216 nm, an x‐axis intercept at 202 nm and a maximum shift at a wavelength 195 nm.^[49]^ No dichroic signals were observed in the 350–500 nm region for Aβ42 alone (Figure 5 inset). For the sake of comparison, the CD spectra of the porphyrin‐peptide conjugates at 20 μM were also recorded in the same experimental conditions, both in the far‐UV and Visible regions (Figure 6S).

**Table 1:** GCI affinity, kinetic and quality parameters.

Ligand	Analyte	K_D_ (μM)	k_on_ (M^‐1^s^‐1^)	k_off_ (s^‐1^)	χ^2[a]^	
A*β*42	MCPTPyP	215±13	1.6±0.2 e^2^	3.5±0.5 e^‐2^	0.63±0.06	
A*β*42	***Pep***	30±7	1.3±0.1 e^2^	3.98±0.8 e^‐2^	0.79±0.08	
A*β*42	***Cu‐Ph‐Pep***	44±6	2.1±0.2 e^3^	9.31±0.6 e^‐2^	0.76±0.07	
A*β*42	***Ph‐Pep***	60±1	1.37±0.01 e^3^	8.19±0.05 e^‐2^	0.71±0.04	
A*β*42	***Zn‐Ph‐Pep***	46±6	1.1±0.2 e^2^	2.0±0.3e^‐3^	0.49±0.09	

[a]χ^2^ is the average of the squared residuals (the average of the squared difference between the measured data points andthe corresponding fitted values) and indicates the fitting confidence.

We then replicated these measurements with the Aβ42/conjugated peptides binary systems. A similar trend, as the measurements carried out at 5 μM, was observed (Figure 5, b–d). Interestingly, in the case of Aβ42/***Zn‐Ph‐Pep*** system an induced dichroic signal, in the porphyrin's Soret absorption region, turned evident with time as the Aβ42 conformational transition toward the β‐sheet in the far UV CD spectra proceeded (see Figure 5, Panel d inset).

To better visualize how the induced signal increases as Aβ42 adopts the β‐sheet conformation, a comparative graph displaying the absolute value of the CD intensities at the maximum absorption wavelength for each derivative, either in the presence or absence of Aβ42 over the same time interval, is reported in the SI (Figure 7S). It is evident that only the ***Zn‐Ph‐Pep*** conjugate generates a distinct CD signal nearly the Soret absorption region. To confirm that the induced CD signal could be correlated with the progressive aggregation of the full‐length of Aβ42, a series of new CD spectra, under the same experimental conditions, were recorded using the Aβ8–20 fragment, whose propensity to aggregation is significantly reduced. (Figure 8S panel a). In our previous work, we demonstrated the ability of the porphyrin amide conjugate Zn‐Porph‐Pep to interact with the Aβ10–20 fragment via NMR analysis.^[31]^ We assume that the conjugate ***Zn‐Ph‐Pep*** conjugate could similarly interact with the Aβ8–20 fragment.

The Far‐UV CD spectra of the Aβ8–20/***Zn‐Ph‐Pep*** mixture also showed curve profiles that suggest the presence of a significant amount of random coil conformation following the interaction with the porphyrin‐peptide conjugate. Consistent with our hypothesis, no induced CD signal is clearly detectable in the visible region. (Figure 8S panel b). The comparative graphic, displaying the absolute value of CD intensities at the maximum absorption wavelength for the ***Zn‐Ph‐Pep*** conjugate, either in the presence or in the absence of Aβ8–20 over the same time interval, is shown in the SI (Figure 9S).

### Neuronal Cytotoxicity

To investigate the potential toxic effect of porphyrin‐peptide conjugates, we evaluated the cell viability by using the MTT assay. We selected the human neuroblastoma cell line, SH‐SY5Y, fully differentiated with all‐trans retinoic acid (RA) to obtain a widely used neuronal‐like model. Interestingly, no toxicity was observed for the analyzed conjugates (Figure 10S) or their appropriate controls (Figure 11S), at all the concentrations used (0.2 μM, 2 μM, 10 μM, 20 μM), after 48 h exposure. The lack of toxicity allowed us to verify whether our porphyrin‐peptide conjugates can enter living cells without explicating severe toxic effects. The obtained biological results are propaedeutic for a future study dealing with in‐cell Aβ detection.

### Live Cell Imaging and Flow Cytometry

The intracellular localization of porphyrins in living cells has already been reported in the literature.[^50^, ^51^] Notably, porphyrins cross the plasma membrane and primarily diffuse into the cytosol. However, depending on the overall charge of the molecule, porphyrin and their conjugates have been shown to associate with membranous organelle such as mitochondria or lysosome.[^50^, ^51^, ^52^] The ability of our compounds to associate with cells or to be taken up by them was investigated by live cell confocal fluorescence and flow cytometry. For this purpose, we exploited the fluorescence of the porphyrin moiety. Fully differentiated SH‐SY5Y cells were exposed to 5 μM of each compound for 24 h, and then fluorescence spectra inside and outside cells were measured as described in the experimental section. The images reported in Figure 6, panel a, show the emission spectra recorded (pixel by pixel) inside cells, compared to the emission spectra recorded for control cells (no porphyrins in the growth medium). A specific porphyrin signal recorded between 600 nm and 700 nm only in the exposed sample, provides evidence that the porphyrin‐conjugated compounds are distributed within the cells. We first measured the emission spectra of each compound to confirm that the fluorescent properties measured *in vitro* were maintained under the conditions applied during the biological assay. Once the specific emission peak typical of porphyrins was detected inside the cells, we measured the average fluorescence intensity for each compound under study. The related histograms are shown in Figure 6, panel c. Although modest, the signal was detected in almost every cell under the microscope stage. Both pristine MCPTPyP and its conjugates here investigated were able to cross the plasma membrane and to distribute within the cells. As reported for other porphyrin‐conjugates in the literature the fluorescent signal was more pronounced in some small areas detected as fluorescent puncta as it is noticeable in Figure 6, panel a. Based on this observation we can speculate that these puncta might correspond to intracellular structures involved in the clearance of potentially toxic compounds. This observation requires further and more in‐depth investigations, as it lays the ground‐work for a promising exploitation of these compounds in the treatment of AD. The results obtained from live cell imaging were confirmed and strengthened by flow cytometry as reported in Figure 6, panel d. As for the live cell imaging, fully differentiated SH SY5Y cells were incubated with 5 μM of each compound and the percentage of fluorescent cells, above the established intensity threshold, was measured after 24 hours, considering a sampled of 20000 cells per condition. As shown in Figure 6, both porphyrin‐conjugates localize within the cell populations as effectively as MCPTPyP does. The large number of cells monitored by flow cytometry provides the statistical significance to support the conclusion that porphyrin‐conjugates are taken up by fully differentiated SH SY5Y cells.

## Discussion

Amyloid peptide's oligomers and fibrils are intensively studied as target for therapy and diagnosis of AD. The early detection of these molecular species is an ambitious goal with numerous studies underway.^[53]^ In this work we demonstrated how the synthesized porphyrin peptide derivatives can interact with the amyloid peptide Aβ42 and influence its aggregation process. It is now well established that the conjugation of biomolecules to porphyrin systems is increasingly being utilized in biomedical applications due to the unique photophysical properties of the tetrapyrrolic macrocycle combined with the versatility of peripheral substituents and metalation state which enable these compounds to target and concentrate within specific biological environments. Although porphyrins, both alone and conjugated with biomolecules, have primarily been used in photodynamic therapy (PDT) applied in the oncology field as well as antibacterial agents,[^22^, ^54^] the application of these systems for the diagnosis and treatment of neurodegenerative diseases has recently gained traction.[^55^, ^56^] For instance, the degradation of Aβ monomer, and related oligomers, has been achieved under photoirradiation in the presence of a porphyrin‐peptide hybrid system containing the KLVFF sequence.^[57]^ As mentioned above, the KLVFF peptide is critical for full‐length Aβ self‐assembly and self‐recognition.[^44^, ^45^, ^58^] The KLVFF mediated interaction with the monomeric and/or aggregated forms of full‐length Aβ underpins the significant antifibrillogenic activity we previously observed with another peptide‐porphyrin derivative.[^31^, ^32^]

Furthermore, our previous work also concerned the synthesis of novel nanomagnets based on Fe_2_O_3_ nanoparticles decorated with amphiphilic cyclodextrins bearing an adamantyl‐conjugated KLVFF oligopeptide at the outer side.^[27]^ The results emphasized the critical role of the KLVFF peptide in conferring these nanomagnets with specific recognition capabilities for the Aβ42 peptide.^[27]^ Assuming that the reported anti‐fibrillogenic ability of the KLVFF‐containing peptide‐porphyrin conjugate relies on direct interaction with Aβ during the early stages of aggregation,[^30^, ^31^] we hypothesized that the presence of the porphyrin chromophore could be exploited to develop a molecular sensor capable of generating an optical signal upon its interaction with the amyloid peptide.

The comparative analysis of the GCI data reported in the present study (see Table 1) confirms the active role of the peptide moiety in the interaction with the amyloid peptide. Taken together, our spectroscopic data suggest that all the derivatives participate in the Aβ42 aggregation process, becoming integrated in the macroaggregate which explains the observed precipitation in the 20 μM mixtures with Aβ42 (not shown). In the literature several examples of fluorescent molecular probes based on functionalized porphyrin have been reported.^[59]^ Interestingly, we observed an induced dichroic activity emerging in the Soret region during the Aβ aggregation in the co‐presence of the ***Zn‐Ph‐Pep*** compound, indicating a potential use of this compound as an Aβ sensor in biological fluids. On the other hand, only the zinc‐containing derivative produced this type of evidence which may be attributed to the zinc ion's ability to apically coordinate with the imidazole side chain of the Aβ42’s histidines, as previously observed.^[31]^ Moreover, the lack of cell toxicity and the ability to penetrate the cells are crucial features of these peptide‐porphyrin conjugates paving the way for their use in detecting of intracellular Aβ peptides in *ex vivo* biological samples including solid or fluid biopsies. So, we can conclude that the careful selection of the peptide chain and linker to the porphyrin macrocycle can lead to unique conjugates with the capability to cross cell membranes and localize specifically within the cells supporting the discovery of novel theragnostic agents and advanced functionalized materials.

## Materials and Methods

### General

Peptide‐synthesis grade N,N‐dimethylformamide (DMF) and HPLC‐grade CH_3_CN were purchased from Sigma‐Aldrich. Rink Amide AM resin, Fmoc‐protected amino acids (Fmoc‐Gly‐OH, Fmoc‐Pro‐OH, Fmoc‐Lys(Boc)‐OH, Fmoc‐Phe‐OH, Fmoc‐Val‐OH, Fmoc‐Leu‐OH) and activators N,N′‐diisopropylcarbodiimide (DIC) and Oxyma pure were purchased from Iris Biotech. Fmoc‐Lys(N3)‐OH was purchased from Sigma‐Aldrich. L‐Propargylglycine methyl ester hydrochloride (L‐Pra‐OMe HCl) was purchased from Chem Impex (USA). Diethyl ether (Et_2_O), copper(II) sulfate pentahydrate (CuSO_4_⋅5H_2_O), Tetrakis(acetonitrile)copper(I)hexafluorophosphate ([(CH_3_CN)_4_Cu]PF_6_, sodium L‐ascorbate, benzotria‐zol‐1‐yloxy(tripyrrolidin‐1‐yl)phosphanium‐hexafluorophosphate (PyBOP), N,N‐Diisopropylethylamine (DIPEA), tert‐butanol (tBuOH), Trifluoroacetic acid (TFA), Triisopropyl silane (TIS), Diisopropyl ether (iPr_2_O), were obtained from Sigma‐Aldrich. 5‐(4‐carboxyphenyl)‐10,15,20‐(tri‐N‐Methyl‐4‐Pyridyl) Porphyrin trichloride (MCPTPyP or ***Ph***) was from Porphychem Company (France). Microwave assisted Solid Phase Peptide Synthesis (MW‐SPPS) was carried out by using an automatic peptide synthesizer Liberty Blue 2.0 (CEM Corporation, Matthews, NC, USA). Analytical and preparative RP‐HPLC were performed using a SHIMADZU LC‐20 A chromatography system equipped with an SPD−M20 A photodiode diode array detector. Detection of the compounds was accomplished at 222, 254, and 400 nm. HPLC eluents were A: 0.1 % TFA/H_2_O and B: 0.1 % TFA/ACN. Fraction contained desired products were concentrated under vacuum, frozen and then lyophilized using a Labconco FreeZone lyophilizer. Mass spectra were recorded on a SCIEX TOF/TOF™ 5800 Matrix‐Assisted Laser Desorption Ionization (MALDI) instrument. The MALDI‐MS spectra were carried out using α‐cyano‐4‐hydroxycinnamic acid (α‐CHCA) as a matrix with thin lay‐er deposition method. 0.1 mg of lyophilized samples were dissolved in 100 μL of 1 : 1:0.01 ACN/H_2_O/TFA. α‐CHCA was prepared dissolving 4 mg/vial of matrices 1 ml of 30 % acetonitrile in 0.3 % TFA. NMR spectra were recorded at 25 °C with a Varian UNITY PLUS‐500 spectrometer. 2D NMR spectra (COSY, TOCSY) were performed using 1 K data points, 256 increments. Spectra were referred to the HDO signal.

### MW‐SPPS of the Azido Peptide Ac‐GK(N3)PGKLVFF‐NH_2_

The peptide was synthesized by a fully automated microwave‐assisted solid phase peptide synthesis (MW‐SPPS) according to the Fmoc/tBu strategy on a Rink amide AM resin (0,22 mmol/g, 450 mg, 100–200 Mesh) in a 0.1 mmol scale. After resin swelling in DMF, Fmoc‐amino acids (Fmoc‐Phe‐OH, Fmoc‐Val‐OH, Fmoc‐Leu‐OH, Fmoc‐Lys(Boc)‐OH, Fmoc‐Gly‐OH, Fmoc‐Pro‐OH and Fmoc‐Lys(N3)‐OH) were introduced through the following protocol: 1) Fmoc‐deprotection in 20 % piperidine in DMF; 2) washes (3×) with DMF; 3) couplings with Fmoc‐amino acid (5 eq, 0.2 M in DMF), Oxyma pure (5 eq, 1 M in DMF) and DIC (5 eq, 0.5 M in DMF) prepared in separated bottles; 4) washes (3×) with DMF. Both deprotection and coupling reactions were performed in a Teflon vessel with microwave energy and nitrogen bubbling. Reaction temperatures were monitored by an internal fiberoptic sensor. The resin was exposed to the microwave‐assisted instrumental parameters indicated in Table 2.

**Table 2:** Instrumental parameters used for the MW‐SPPS.

Step	Temperature (°C)	Power (W)	Time (s)
**Deprotection**	75	175	15
	90	37	50
	65	220	30
**Coupling**	75	175	15
	90	55	110

Final cleavage from the resin with concomitant sidechains deprotection was achieved by treatment of resin‐bound peptide with a TFA/TIS/H_2_O solution (95 : 5 : 5, 1 mL mixture/100 mg of resin). The cleavage was carried out for approximately 3 h at room temperature. The resin was filtered and then rinsed with TFA (2×1 mL). The peptide solution was added to the washes and the product was precipitated from this solution by the addition of ice‐cold Et_2_O (30 mL). The precipitated material was washed with ice‐cold Et_2_O (3×3 mL) and dried under vacuum. The solid peptide was then dissolved in H_2_O (5 mL), iced at −30 °C overnight and lyophilized. The crude peptide was purified by RP‐HPLC using a Jupiter C_12_ 250×21, 2 mm (300 Å pore size, AXIA Packet) preparative column. The peptide was eluted at a flow rate 10 ml/min by the following gradient: from 0–5 min isocratic elution with 90 % A, then linear gradient from 10 %–80 % B in 15 min, finally isocratic elution with 80 % B; Rt=18.1 min. The peptide identity was determined by MALDI‐TOF MS [obsd: m/z (M+H)^+^=1059,91; (M+Na)^+^=1081,908; (M+K)^+^=1097,89. calcd. for C_52_H_78_N_14_O_10_: 1059.20].

### Synthesis of (4‐(10,15,20‐Tris(1‐Methyl‐Pyridin‐4‐Yl)Porphyrin‐5‐Yl)Benzoyl)Propargylglycine Methyl Ester (Ph‐Pra‐OMe)

To a solution of 5‐(4‐carboxyphenyl)‐10,15,20‐(tri‐N‐methyl‐4‐pyridyl)porphyrin trichloride (Ph) (40 mg, 0.05 mmol) in DMF (2 ml), a mixture of PyBOP (85.8 mg, 3,3 eq), HOBt (22.3 mg, 3,3 eq), DIPEA (57.5 μL, 6,6 eq) and L‐propargylglycine methyl ester (Pra‐OMe) (81.8 mg, 10 eq) in DMF (3 ml) was added and the reaction mixture was stirred at room temperature for 3 h. The solution was concentrated under vacuum and the crude product was purified via preparative RP‐HPLC using a Jupiter C_4_ 250×21, 2 mm (300 Å pore size, AXIA Packet) column. The product Ph‐Pra‐OMe was eluted at a flow rate 10 ml/min by the following gradient: from 0–5 min isocratic elution with 10 % B, then linear gradient from 10 %–55 % B in 15 min, finally isocratic elution with 55 % B; Rt=16 min.

### Porphyrin Metalation with Cu(II) of Ph‐Pra‐OMe to Obtain Cu‐Ph‐Pra‐OMe

A solution of Ph‐Pra‐OMe (30 mg, 0.036 mmol) in THF/Acetic Acid 1 : 1 (4 ml) was treated with Cu(CH_3_COO)_2_ (65.4 mg, 10 eq) and the mixture was stirred at room temperature, overnight. The metalated conjugate Cu−Ph‐Pra‐OMe was concentrated under vacuum and directly purified by RP‐HPLC using a Jupiter C_12_ 250×21, 2 mm (300 Å pore size, AXIA Packet) column. HPLC method from 0–3 min isocratic elution with 0 % B, then linear gradient from 0 %–55 % B in 7 min, finally isocratic elution with 55 % B, flow rate 10 ml/min; Rt=19 min.

### Conjugation of Cu‐Ph‐Pra‐OMe with Ac‐GK(N3)PGKLVFF‐NH_2_ by Azide‐Alkyne Click Reaction in Solution to Obtain *Cu‐Ph‐Pep*

A solution of Cu−Ph‐Pra‐OMe (20 mg, 0.023 mmol) and azido peptide Ac‐GK(N3)KLVFF‐NH_2_ (48.7 mg, 2 eq) in DMF (5 ml) was treated with CuSO_4_⋅5H_2_O (7.5 mg, 2 eq) reduced to Cu(I) with ascorbic acid (40.5 mg, 10 eq). The click reaction was carried out at 50 °C, for 24 h and at room temperature for 3 days and was monitored by analytical HPLC using a Jupiter C_12_ 250×4, 6 mm (Proteo 90 Å pore size) column at a flow rate 1,25 ml/min by the following gradient: from 0–5 min isocratic elution with 10 % B, then linear gradient from 10 %–70 % B in 10 min, finally isocratic elution with 80 % B; Rt Cu−Ph‐Pra‐OMe=16, 1 min, Rt (Ac‐GK(N3)KLVFF‐NH_2_)=17 min, Rt ***Cu‐Ph‐Pep*** =16, 5 min. After completion of the reaction the solvent was removed under vacuum and the desired peptide conjugate purified with preparative HPLC using a Jupiter C_4_ 250×21, 2 mm (300 Å pore size, AXIA Packet) column. ***Cu‐Ph‐Pep*** was eluted at a flow rate 10 ml/min by the following gradient: from 0–5 min isocratic elution with 10 % B, then linear gradient from 10 %–55 % B in 10 min, finally isocratic elution with 70 % B; Rt=15, 8 min. The ***Cu‐Ph‐Pep*** identity was determined by MALDI‐TOF MS [obsd: m/z (M+H)^+^=1937, 73; calcd. for C_103_H_118_CuN_22_O_13_: 1936,77].

### Conjugation of Ac‐GK(N3)PGKLVFF‐NH_2_ with Pra‐OMe by Azide‐Alkyne Click Chemistry on‐Resin

After swelling of the precursor azido peptide‐resin Ac‐GK(N3)PGK(Boc)LVFF‐Rink Amide AM resin (57 mg, 0.01 mmol) in DMF (3 ml), L‐Pra‐OMe HCl (3.2 mg, 2 eq), [(CH_3_CN)_4_Cu]PF_6_ (5,6 mg, 1,5 eq) Ascorbic Ac‐id(17.6 mg, 10 eq) and DIEA (17.5 μL, 10 eq) was added and the mixture was stirred at 37 °C overnight to obtain the on resin intermediate Ac‐GK(1,2,3‐triazol‐4‐yl)Ala‐OMe)PGK(Boc)LVFF‐Rink Amide AM resin.

### On Resin Porphyrin Conjugation and Cleavage from the Resin to Obtain *Ph‐Pep*

After swelling of the Ac‐GK(1,2,3‐triazol‐4‐yl)Ala‐OMe)PGK(Boc)LVFF‐Rink Amide AM resin (65 mg, 0.01 mmol) in DMF (3 ml), a solution of Ph (20.3 mg, 2.5 eq)/DIC (4 ml, 2.5 eq)/Oxyma (3.5 mg, 2,5 eq) in DMF (1 ml) was added. The porphyrin conjugation was carried out for 72 h at 37 °C. The reagents in excess were removed by filtration and the resin was washed twice with DMF (3 ml), H_2_O (3 ml), DMF (3 ml) and CH_2_Cl_2_ (3 ml). The peptide unmetalated porphyrin‐peptide ***Ph‐Pep*** was obtained after cleavage from the resin (as described above) by a mixture of TFA/H_2_O/TIS (3 ml, 95 : 2.5 : 2.5 v/v/v), precipitated in cold diethyl ether (20 ml), and lyophilized. The ***Ph‐Pep*** identity was determined by MALDI‐TOF MS [obsd: m/z (M+H)^+^=1876,12; calcd. for C_103_H_121_N_22_O_13_: 1875,24]. The purified ***Ph‐Pep*** was further characterized by Cosy and Tocsy 2D‐NMR (see supplementary material Figures 12S and 13S, and Table 1S).

### Zinc(II) Metalation to Obtain *Zn‐Ph‐Pep*

The conjugate ***Ph‐Pep*** was dissolved in THF/Acetic Acid 1 : 1 (4 ml) and treated with Zn(CH_3_COO)_2_ (20 mg, 10 eq). The mixture was stirred at room temperature, overnight, concentrated under vacuum and directly purified by RP‐HPLC using a Jupiter C_4_ 250×21, 2 mm (300 Å pore size, AXIA Packet) column. ***Zn‐Ph‐Pep*** was eluted at a flow rate 10 ml/min by the following gradient: from 0–5 min isocratic elution with 10 % B, then linear gradient from 10 %–55 % B in 10 min, finally isocratic elution with 70 % B; Rt=15, 8 min. the identity of ***Zn‐Ph‐Pep*** was confirmed by MALDI‐TOF MS [obsd: m/z (M+H)^+^=1939, 43; calcd. for C_103_H_119_ZnN_22_O_13_: 1938,60].

### Sample Preparation

The necessary condition to obtain a reliable reproducibility in all the experiment accomplished with amyloid systems is hampered because of their high tendency to self‐aggregating. To remove any preexisting aggregated forms, we applied a previously standardized monomerization procedure to treat Aβ42 as well as all the tested peptides in this work.^[25]^ The peptide was dissolved in trifluoro acetic acid (TFA) (1 mg/ml) and sonicated in a water bath sonicator for 10 min. Then the TFA was evaporated under a N_2_ gentle stream to obtain a thin film adhering the wall vessel. In the case of Porphyrin‐conjugate the first step of monomerization with TFA was removed. Subsequently 1 ml of 1,1,1,3,3,3‐hexafluoro‐2‐propanol (HFIP) was added to the peptide. HFIP is well known for its ability to stabilize local hydrogen bonds between residues close in the amino‐acid sequence, particularly those forming α‐helices, it can disrupt the β‐conformation by destabilizing hydrophobic interactions.^[60]^ After 1 h incubation at 37 °C, the peptide solution was dried under a N_2_ stream, the peptide film was dissolved in 2 ml HFIP, dried under a N_2_ stream to remove remaining trace of TFA, again dissolved in 1 ml HFIP and frozen at −80 °C for 4 or 5 hours, then lyophilized overnight. The lyophilized Aβ42 samples were dissolved in NaOH 20 mM (2,5 % total volume) and after was diluted with 1950 μL of 10 mM phosphate buffer pH 7.4 pure or containing the solubilized conjugates. In the case of biological experiments, the lyophilized sample was dissolved in Dimethylsulfoxide (DMSO) at a 5 mM concentration stock solution.

### UV‐Vis

Spectra were recorded on a Jasco V‐670 Spectrophotometer at room temperature. All measurements were performed in the 300–650 nm wavelength region, in a 10 mM phosphate buffer solution at pH 7.4 using a 1 cm path length quartz cell. The spectra of all the peptide‐porphyrin conjugates, either in the absence or in the presence of equimolar Aβ42, were acquired at 5 μM. In some cases, the same sample solution was used to run the CD spectra. The final concentrations of Aβ42 and peptide conjugates were 5 μM (1 : 1 molar ratio).

### Circular Dichroism (CD)

Spectra were recorded on a J‐810 spectrometer (Jasco, Japan) under a constant flow of N_2_ at room temperature. The CD measurements were performed in the wavelength regions (190–260 nm, 195–350 nm and 350–500 nm) using a 1 cm path length quartz cell. All aggregation kinetics were monitored up to 120 hours. The CD spectra were recorded for Aβ42 (5 μM and 20 μM) monomer in the absence or in the presence of porphyrin‐peptide conjugates (5 μM and 20 μM for the 1 : 1 molar ratios). Monomerized samples of Aβ42 (5 μM and 20 μM) were solubilized in 50 μL of NaOH 20 mM (2,5 %) and after were diluted with 1950 μL of 10 mM phosphate buffer at pH 7.4 pure or containing the solubilized conjugates.

### Fluorescence

Spectra were recorded on a Horiba Fluoro‐max‐4 spectrofluorometer from 440–800 nm in 1 nm steps, at an excitation wavelength λ_exc_=425 nm, at room temperature. The final fluorescence spectra represent averages of at least three measurements. Bandwidths of 5 nm for both excitation and emission were used. The sample of the compound used to record absorption spectra were the same as those for the CD spectra.

### Bis‐Ans Fluorescence

Bis‐Ans fluorescence kinetics were measured on a Flash Thermo Varioskan spectrofluorometer with excitation and emission at 385 and 510 nm, respectively. Aβ42, alone or in the presence of the porphyrin‐peptide conjugates in a 1 : 1 molar ratio, was dissolved in 10 mM aq NaOH (30 μL). The samples were diluted (to 150 μL) with 60 μM Bis‐Ans solution in 10 mM phosphate buffer at pH 7.4 to obtain a final Aβ42 concentration of 20 μM. The samples were incubated a 37 °C in a 96‐wells plate. To minimize evaporation effects the multiwall plate was sealed with a transparent heat‐resistant plastic film. Readings were taken every 10 min, after weak shaking for 10 s. The fluorescence intensity was monitored for 65 h. The measurements were performed in triplicate.

### Grating‐Coupled Interferometry (GCI)

The experiments were performed on a Creoptix WAVE system (Creoptix). Borate buffer (100 mM sodium borate pH 9.0, 1 M NaCl) was used for chip conditioning. Aβ42 peptide (20 μg/ml in Na Acetate pH 4.5) was immobilized on 4PCH WAVE chips (quasiplanar polycarboxylate surface; Creoptix) according to the manufacturer instructions at a final density of 1910±40 pg/mm^2^. Single‐cycle sequential injections of dilution series for each Porphyrin‐Peptide conjugates in running buffer (PBS, 0.005 % Tween 20, 1 % DMSO, 0.2 mg/ml BSA) were performed at 25 °C, using a flow rate of 100 μl/min. In details, for the conjugate ***Cu‐Ph‐Pep***, 1 : 2 dilution series starting from 200 μM were injected for 10 s association time, 45 s dissociation time, followed by a dissociation time of 600 s for the last higher concentration injection. Finally, for the conjugates ***Ph‐Pep*** and ***Zn‐Ph‐Pep***, 1 : 2 dilution series starting from 50 μM and 100 uM, respectively, were injected for 30 s association time, 45 s dissociation time, followed by a dissociation time of 600 s for the last higher concentration injection. Blank injections were used for double referencing and a DMSO calibration curve for bulk correction. Analysis and correction of the obtained data were performed using the Creoptix WAVE control software (correction applied: X and Y offset; DMSO calibration; double referencing). Mass transport binding models with bulk correction were used to fit all experiments. All the experiments have been performed at least in triplicate.

### Cell Cultures and MTT Assay

The neuroblastoma cell line, SH‐SY5Y, was maintained in DMEM−F12 (Gibco, ThermoFisher) supplemented with 10 % heat‐inactivated (HI) fetal calf serum (Gibco, ThermoFisher), 100 mg/mL penicillin and streptomycin (Gibco, ThermoFisher), and 2 mM L‐glutamine at 37 °C, 5 % CO_2_. Two weeks before experiments, 5×10^3^ cells were plated on 96‐well plates in DMEM−F12 with 5 % HI fetal calf serum. The percentage of serum was gradually decreased until it was 1 % of the total. All‐trans‐retinoic acid (RA) (Sigma), 5 μM, was used to promote neuronal differentiation, and medium‐containing RA was changed every 3 days. Fully differentiated SH‐SY5Y cells were treated with increasing concentrations (0,2 μM, 2 μM, 10 μM and 20 μM) of all the porphyrin‐peptide conjugates tested and the relative controls (***Ph***, Ac‐GK(N3)PGKLVFF‐NH_2_). 48 h later, cultures were incubated with MTT (5 mg/mL) for 2 h at 37 °C, lysed in dimethyl sulfoxide (DMSO), and the amount of formazan was evaluated by reading the absorbance at 570 nm using a Varioskan multimode reader.

### Flow Cytometry

Two weeks before experiments, human SH‐SY5Y cells were seeded on a 12‐well plate at a density of 7×10^4^ and differentiated upon RA, as described above. Fully differentiated SH‐SY5Y cells were exposed to 5 μM of Porphyrin‐conjugated peptides. 24 h later, cells were harvested, resuspended in cold Phosphate‐buffered saline (PBS) (Gibco, ThermoFisher) and then analyzed by using a CyFlow® ML flow cytometer (Partec) system, equipped with three laser sources and 10 optical parameters with dedicated filter setting and a high numerical aperture microscope objective (50×NA 0.82) for the detection of different scatter and fluorescence signals. Sample gating, based on forward and side scatter of 4×104 cells, was used for analysis. Fluorescent signals of porphyrin‐conjugated peptides were measured at single cells levels by exciting with an UV 375 laser and reading the signal emitted on FL4 log mode. The percentage of fluorescent cells in FL4 was determined by fixing a fluorescence threshold based on the difference between negative (untreated cells) and positive (porphyrins treated) controls. Data obtained were acquired and gated using the FlowMax software (Sysmex Partec, Milan, Italy). Histograms reported were obtained from three sets of independent experiments, each performed in triplicate and based on 40.000 events for each sample. Analysis and graphical representation were obtained by using the FCS express 5 software.

### Live Cell Imaging

Human neuroblastoma SH‐SY5Y cells were plated on chambered coverslips (μ‐Slide 8 Well Glass Bottom, Ibidi, Gräfelfing, MU, Germany) at a density of 1×10^4^ and allowed to differentiate as above described. Differentiated SHSY5Y were exposed to 5 μM of ***Ph‐Pep***, ***Zn‐Ph‐Pep*** and ***Ph*** for 24 h and imaged under a Leica TCS SP8 confocal microscope, using an HC PL APO CS2 20X 0.75 NA objective lens and two hybrid photodetectors (Leica Microsystems, Mannheim, BW, Germany). Live cells imaging was performed by incubating samples on the confocal stage at 37 °C, 5 % CO_2_ for a total of 1 h. Based on fluorescence spectra measured for each compound in solution (in absence of cells), samples were excited with the 405 nm laser and the emissions spectra for each compound were collected by using the lambda scan function. Since specifical signal were detected for all sample between 600 nm and 700 nm, all micrographs were acquired using 405 nm excitation wavelengths/600–700 nm emission bandwidths. The pinhole size was set to a value corresponding to 1 Airy Unit. Then, 2048×2048‐pixel images were acquired with a pixel size of 189 nm. Transmitted light images showing the morphology of the cells were acquired using the transmitted light detector (TLD) during excitation with the 488 nm laser. Acquisition parameters were kept constants for all the samples. The fluorescence intensity was quantified considering 10 regions of interest (ROI) inside each cell and extracting the average ROI value after background subtraction. For each experimental condition, a minimum of 10 cells, 3 independent images obtained by 2 independent experiments were acquired and analyzed.

## Conflict of Interests

The authors declare no conflict of interest.

1

## Supporting information

As a service to our authors and readers, this journal provides supporting information supplied by the authors. Such materials are peer reviewed and may be re‐organized for online delivery, but are not copy‐edited or typeset. Technical support issues arising from supporting information (other than missing files) should be addressed to the authors.

Supporting Information

## Data Availability

The data that support the findings of this study are available in the supplementary material of this article.
