# Supplementary material for: A Spectroscopic Study on the Amyloid‐β Interaction with Clicked Peptide‐Porphyrin Conjugates: a Vision Toward the Detection of Aβ Peptides in Aqueous Solution
Source: Chembiochem. 2024 Nov 7;25(23):e202400431. doi: 10.1002/cbic.202400431 (PMC11610669; doi:10.1002/cbic.202400431)
Supplement: Supplementary file 1 — Supporting Information [file CBIC-25-e202400431-s001.pdf]

ChemBioChem

Supporting Information

A Spectroscopic Study on the Amyloid-β Interaction with Clicked Peptide-Porphyrin Conjugates: a Vision Toward the Detection of Aβ Peptides in Aqueous Solution

Rita Tosto, Stefania Zimbone, Giuseppina Sabatino, Giuseppe Di Natale, Maria Laura Giuffrida, Marianna Flora Tomasello, Luca Lanzanò, Tiziana Campagna, Sonia Covaceuszach, Graziella Vecchio, and Giuseppe Pappalardo*

SUPPORTING INFORMATION

A spectroscopic study on the Amyloid-β interaction with clicked peptide-porphyrin conjugates: a vision toward the detection of Aβ peptides in aqueous solution.

Rita Tosto,[1,2] Stefania Zimbone,[1] Giuseppina Sabatino,[1] Giuseppe Di Natale,[1] Maria Laura Giuffrida,[1] Marianna Flora Tomasello,[1] Luca Lanzanò, Tiziana Campagna,[1] Sonia Covaceuszach,[4] Graziella Vecchio,[5] Giuseppe Pappalardo. [1*]

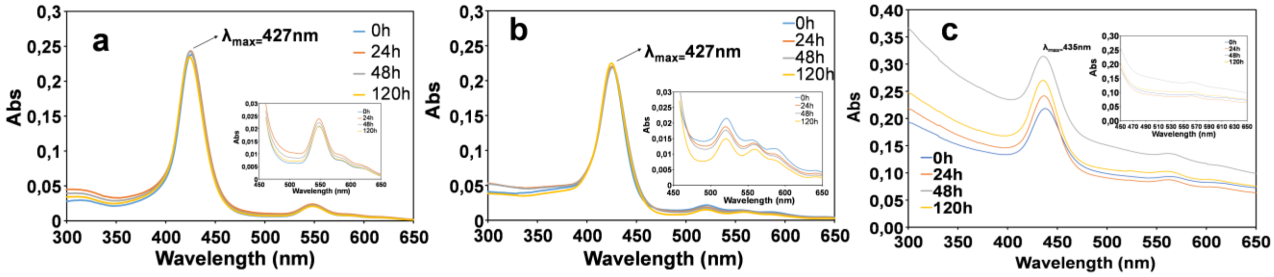

Figure 1S. UV-Vis spectra acquired at different time intervals, in the region between 300-650 nm of a) ***Cu-Ph-Pep***, b) ***Ph-Pep***, c) ***Zn-Ph-Pep***.

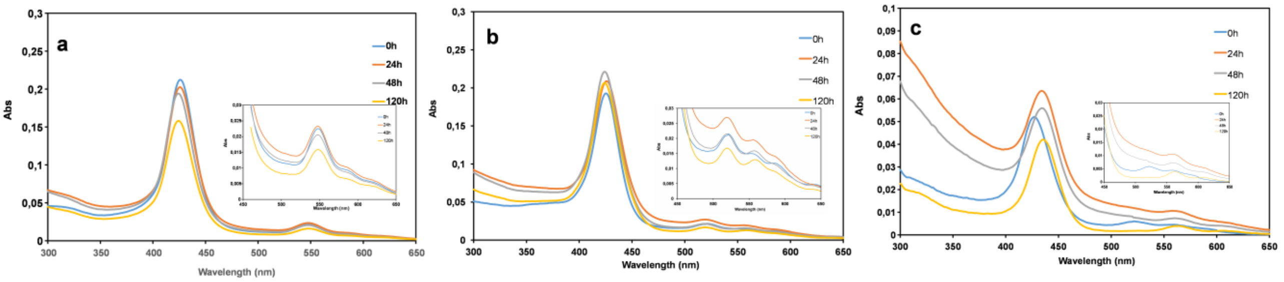

Figure 2S. UV-Vis spectra of porphyrin-peptide conjugates recorded in presence of Aβ_{42} monomers, a) Aβ_{42}/***Cu-Ph-Pep;*** b) Aβ_{42}/***Ph-Pep***; c) Aβ_{42}/***Zn-Ph-Pep***.

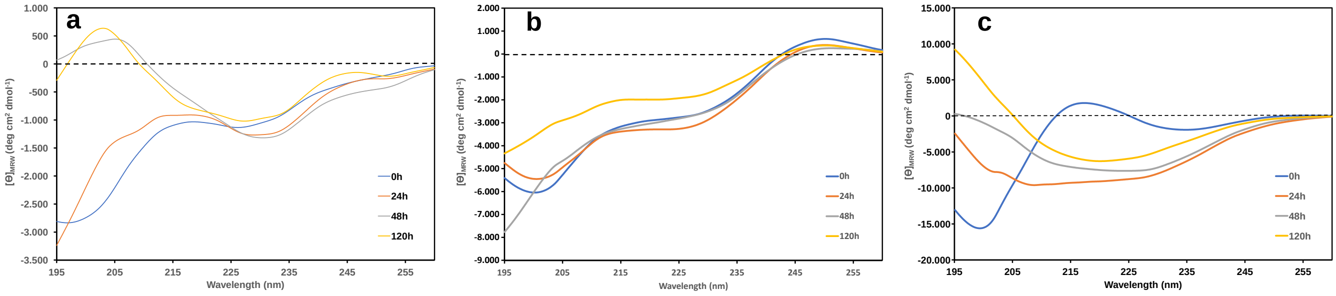

Figure 3S. CD spectra of conjugates at 5µM concentration recorded at different time intervals a) ***Cu-Ph-Pep***; b) ***Ph-Pep***; and c) ***Zn-Ph-Pep.***

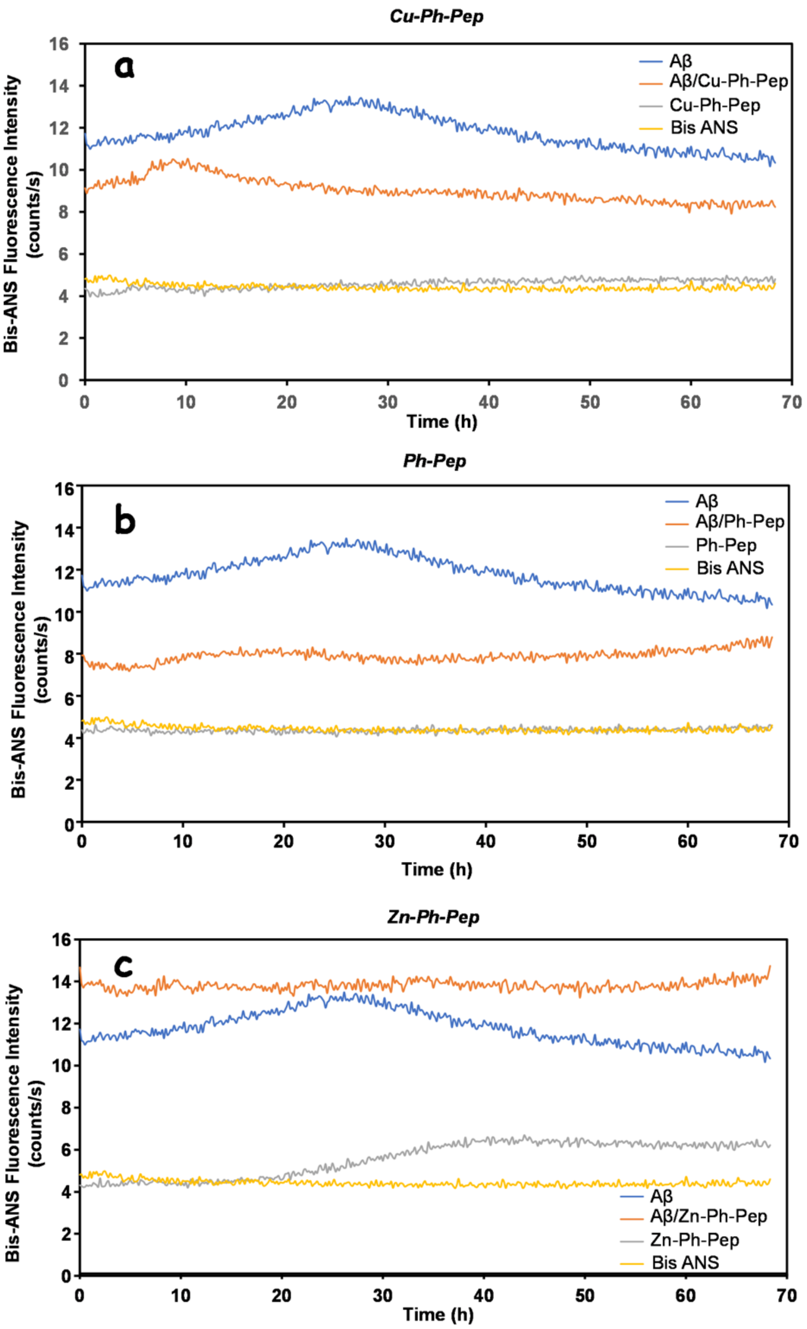

Figure 4S. bis-Ans fluorescence kinetics of $A\beta_{42}$ (20 µM), $A\beta_{42}$/porphyrin-peptide conjugates at a 1:1 molar ratio and peptide conjugates alone. a) ***Cu-Ph-Pep***; b) ***Ph-Pep***; c) ***Zn-Ph-Pep.***

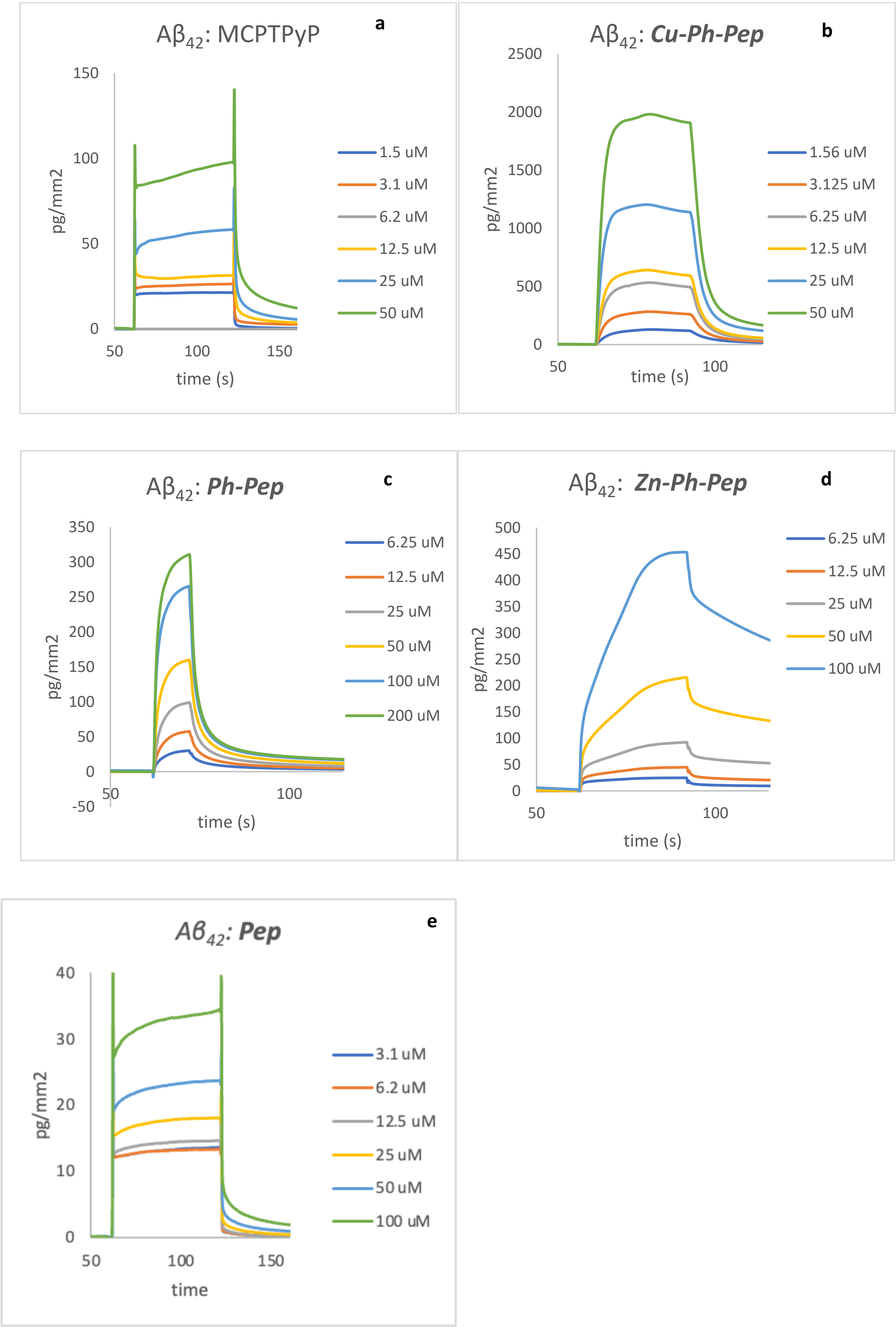
Aβ42: MCPTPyP
a
150
100
50
0
pg/mm2
50
100
150
time (s)
1.5 uM
3.1 uM
6.2 uM
12.5 uM
25 uM
50 uM
Aβ42: Cu-Ph-Pep
b
2500
2000
1500
1000
500
0
pg/mm2
50
100
time (s)
1.56 uM
3.125 uM
6.25 uM
12.5 uM
25 uM
50 uM
Aβ42: Ph-Pep
c
350
300
250
200
150
100
50
0
-50
pg/mm2
50
100
time (s)
6.25 uM
12.5 uM
25 uM
50 uM
100 uM
200 uM
Aβ42: Zn-Ph-Pep
d
500
450
400
350
300
250
200
150
100
50
0
pg/mm2
50
100
time (s)
6.25 uM
12.5 uM
25 uM
50 uM
100 uM
Aβ42: Pep
e
40
30
20
10
0
pg/mm2
50
100
150
time
3.1 uM
6.2 uM
12.5 uM
25 uM
50 uM
100 uM

Figure 5S. Quantitative binding kinetics of Aβ_{42} vs a) MCPTPyP; b) ***Cu-Ph-Pep***; c) ***Ph-Pep***; d) ***Zn-Ph-Pep***; and e) ***Pep*** measured by GCI. All curves were blank subtracted. Experiments were performed in triplicate. All the quality assessments (i.e., $\chi 2$ shown in Table 1 of the main text, parameter errors, and residual plots were acceptable; the sensorgrams had sufficient curvatures and the kinetic constant k_{off} were within the measurable range) were fulfilled. Descriptive Statistical Analysis was employed to calculate the mean of the obtained values of k_{on}, k_{off} and K_D as a measure of central tendency and standard deviations of the former values were computed as a measure of dispersion.

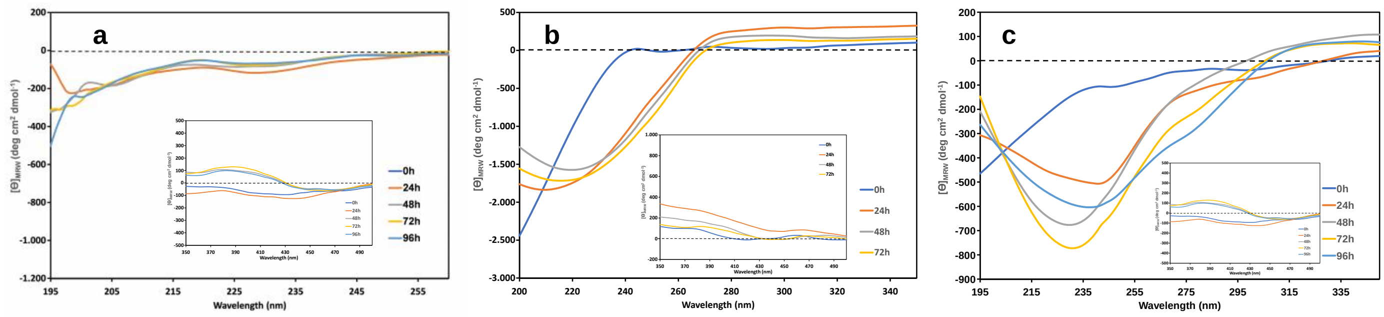

Figure 6S. CD Spectra the porphyrin-peptide conjugates at 20 µM in the Far-UV region (195-350 nm). **a**) **Cu-Ph-Pep**; **b**) **Ph-Pep**; **c**) **Zn-Ph-Pep**. inset: CD spectra in the near UV region (350-500 nm).

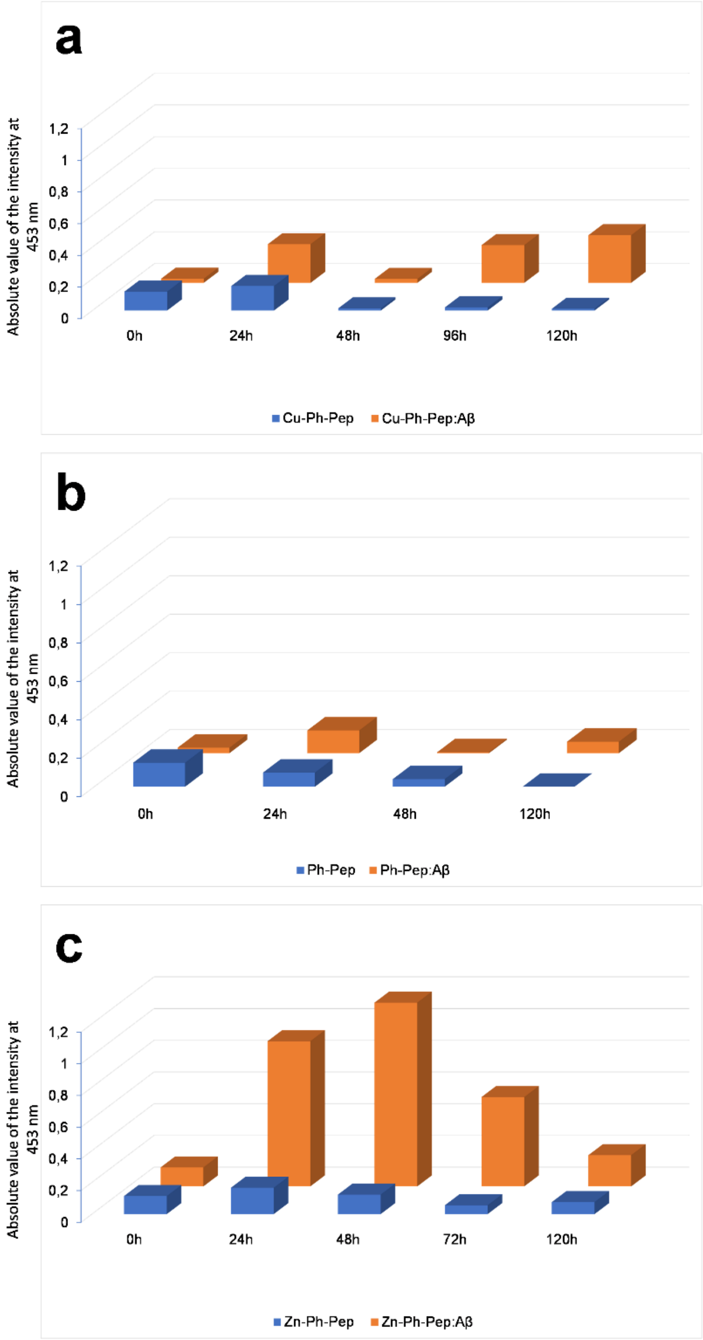

Figure 7S. Comparative graphic showing the absolute value of CD intensities at maximum absorption wavelength for each peptide derivative either in the presence or absence of $A\beta_{42}$ at the same interval of time.

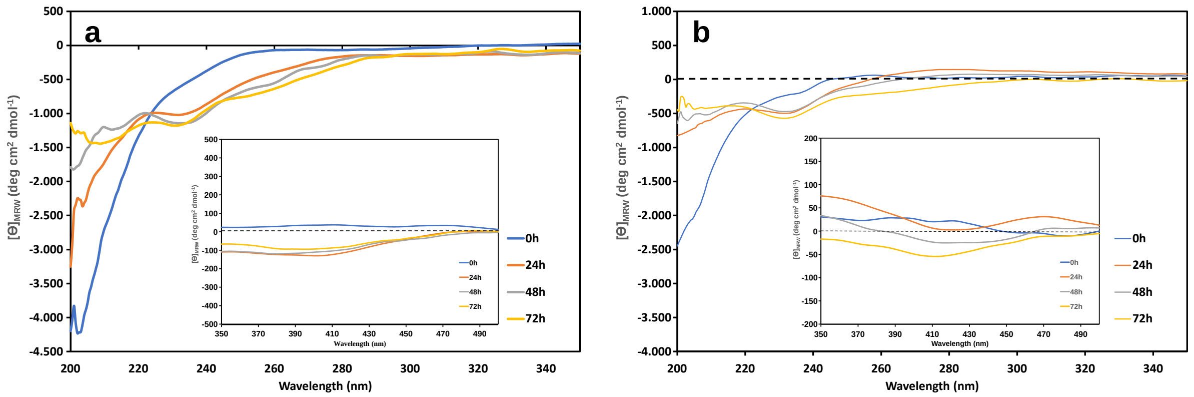

Figure 8S. Panel **a**: CD Spectra of Aβ $_{8\text{-}20}$ (20 µM) in the Far-UV (195-350 nm) and visible (350-500 nm, inset) regions**.** Panel **b**: CD Spectra of Aβ $_{8\text{-}20}$ /***Zn-Ph-Pep*** equimolar mixture (20 µM) in the Far-UV (195-350) and near-UV (350-500 nm) regions.

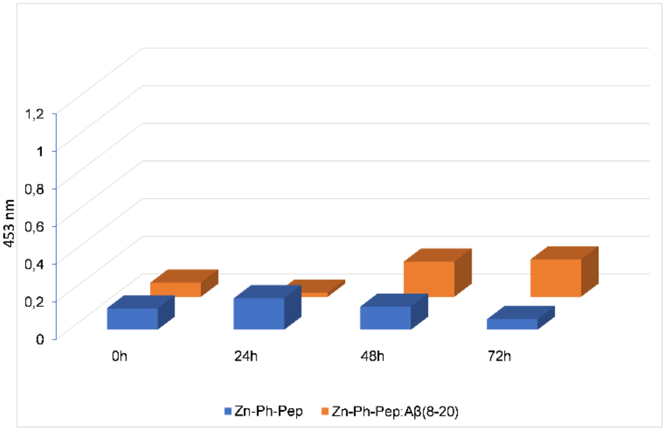

Figure 9S. Comparative graphic showing the absolute value of CD intensities at maximum absorption wavelength for conjugate ***Zn-Ph-Pep*** either in the presence or absence of Aβ $_{8\text{-}20}$ at the same time interval.

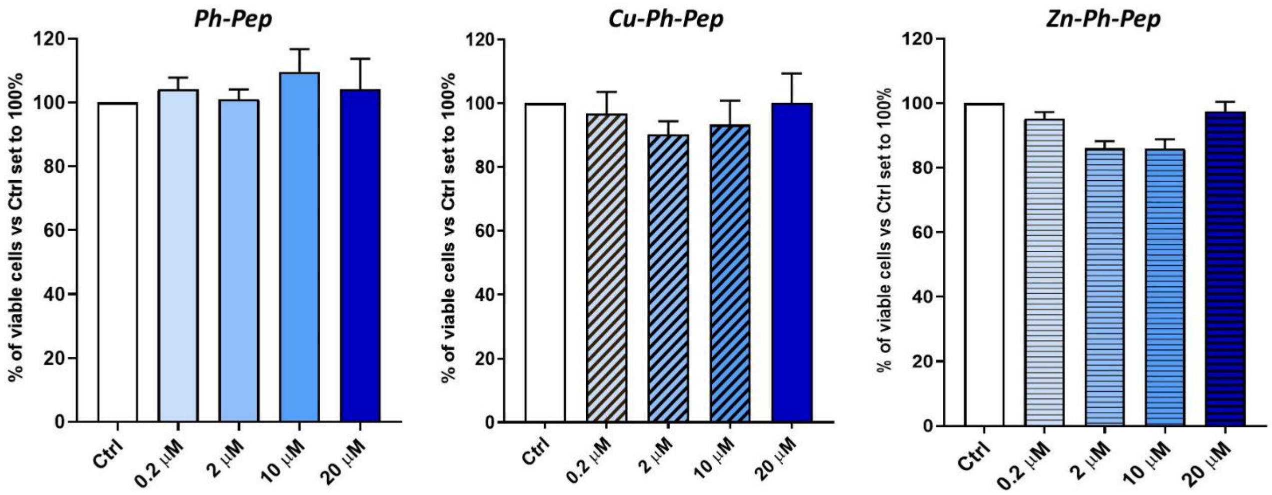

Figure 10 S. MTT Assay of fully differentiated SH-SY5Y treated for 48 hours with increasing concentrations of ***Ph-Pep*** and its derivatives (0.2, 2, 10, 20 µM). Bars represent means ±SEM of three independent experiments with n=3 each.

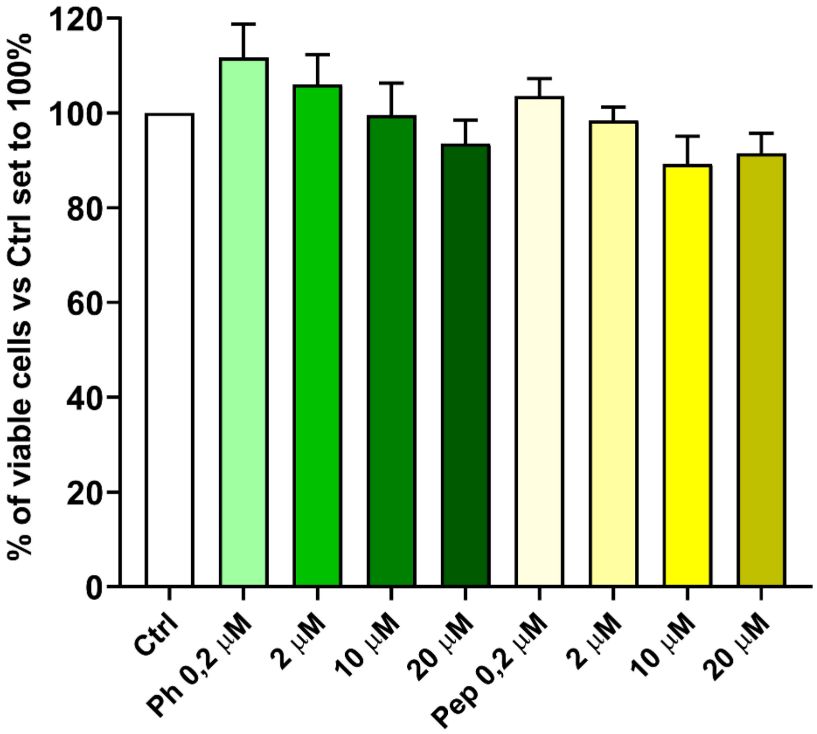

Figure 11S. MTT Assay of fully differentiated SH-SY5Y treated for 48 hours with increasing concentrations of pristine MCPTPyP (**Ph**)and Ac-GK(N_3)PGKLVFF-NH_2 Peptide (Pep) (0.2, 2, 10, 20 µM), separately added to evaluate the potential toxicity of the single components of the compounds. Bars represent means ±SEM of three independent experiments with n=3 each.

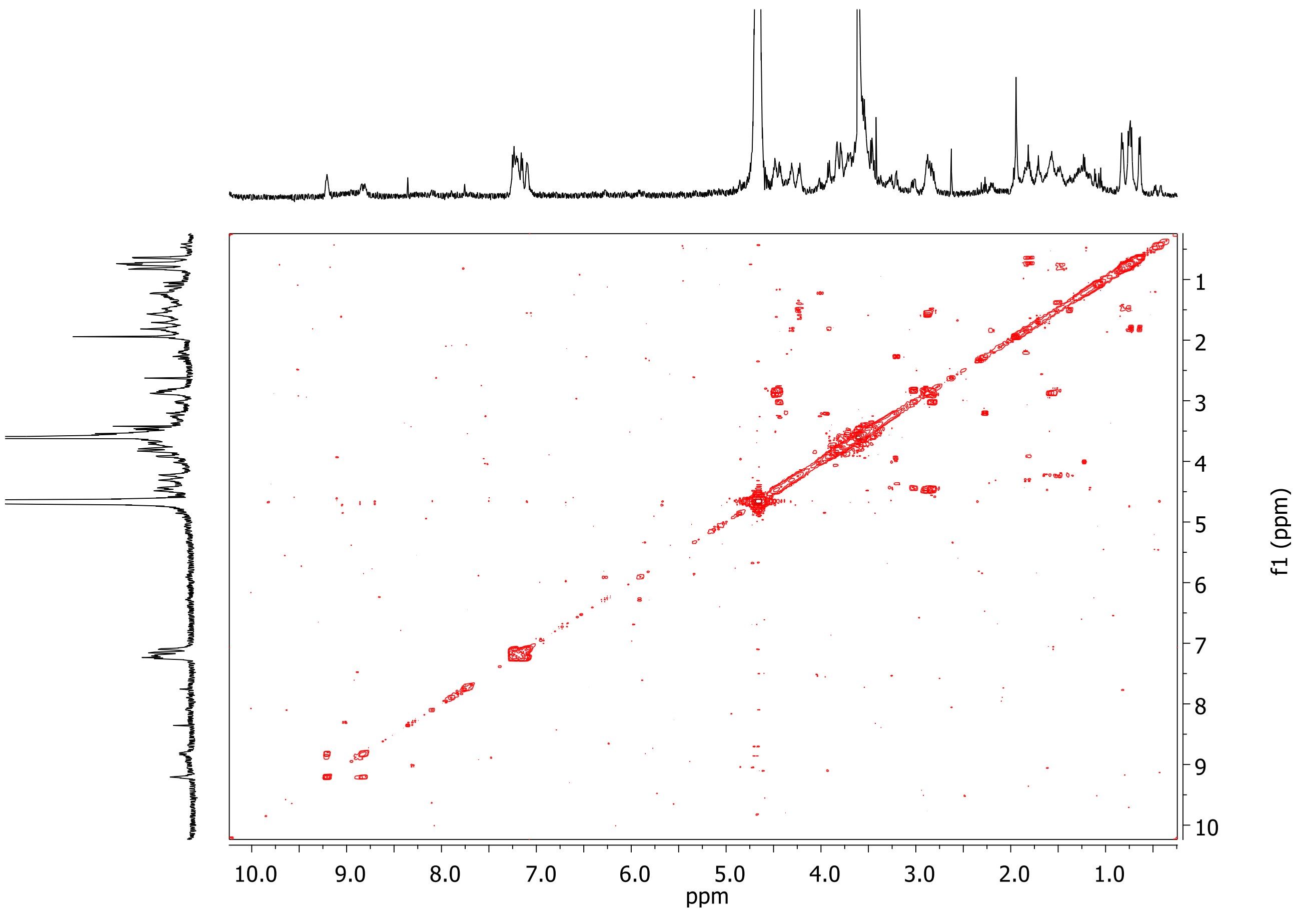

Figure 12S 2D COSY ***Ph-Pep***

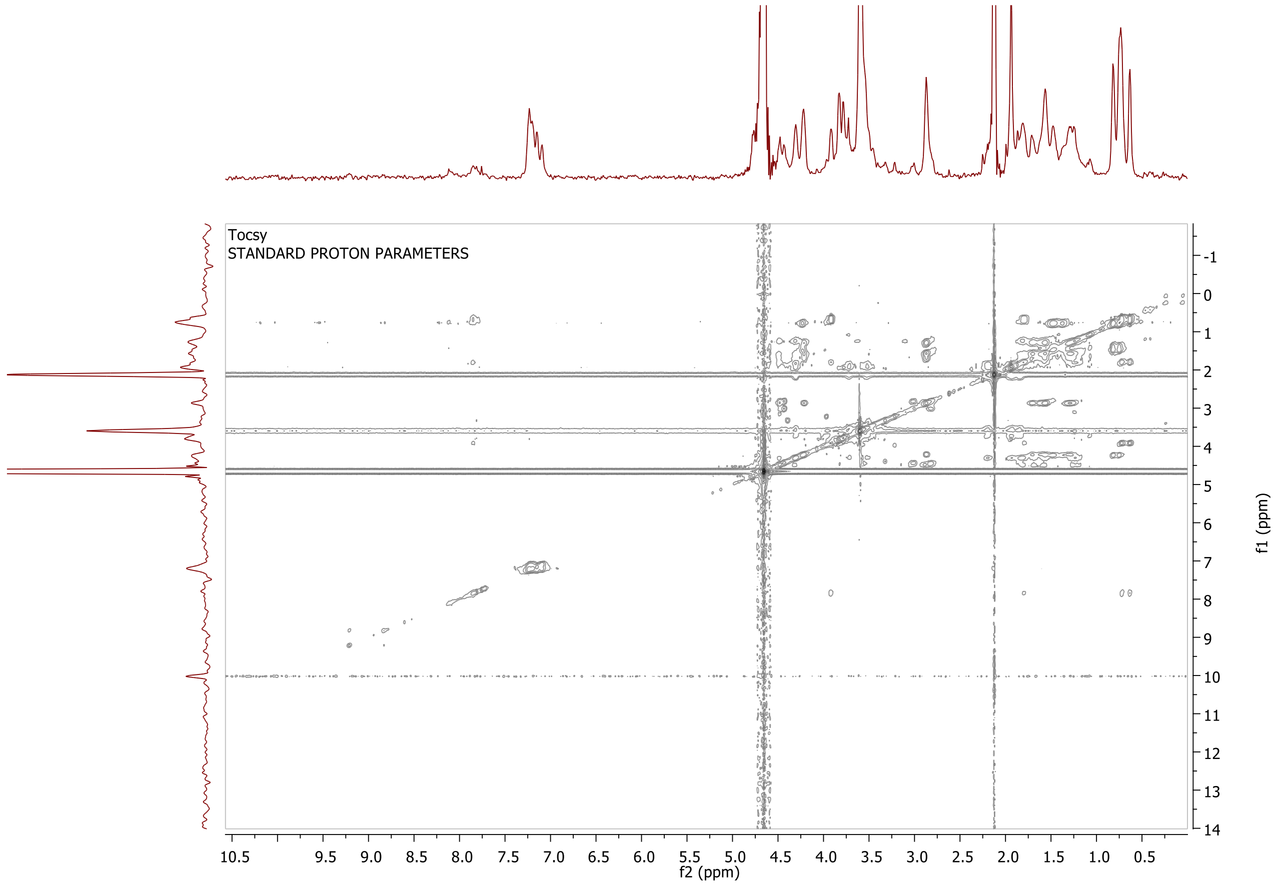

Figure 13S 2D TOCSY ***Ph-Pep***

Table 1S. Proton Chemical shifts (ppm) for the peptide-conjugate ***Ph-Pep***.

Position	Hα	Hβ	Hγ	Others
(1,2,3-triazol-4-yl)AlaOMe	3.89;	3.79; 3.75		O-CH_3 3.72
Lys(N_3)	4.23	1.61; 1.58	1.31	δCH_2 1.68; εCH_2 2.90
Gly(Ac)*	3.83;3.78			CH_3 1.95
Pro	4.31	2.21; 1.83	1.94	δCH_2 3.54;3.71
Gly*	3.34; 3.71			
Lys	4.48	1.69;1.58	1.35	δ$CH_2$1.68; ε$CH_2$3.05
Leu	4.23	1.50	1.49	$CH_3$0.81; $CH_3$0.75
Val	3.93	1.80	CH_3 0.72; $CH_3$0 .64	
Phe	4.43	3.03; 2.84		ar7.09; ar7,22
Phe	4.48	2.82; 2.91		ar7.09; ar7.22
MCPTPyP mojety				
Piridyl$_{2,6}$ 9.18	Piridyl $_{3,5}$ 8.18	Pirrolyl $_{3,4}$ 8.93; 8.72	Benzyl $_{2,6}$; 7.17 Benzyl $_{3,5}$ 6.51	N-CH_3 3,61
